# For the Sake of Production—And the Animal, and Me. How Students at Danish Agricultural Colleges Perceive Animal Welfare

**DOI:** 10.3390/ani11030696

**Published:** 2021-03-05

**Authors:** Inger Anneberg, Jesper Lassen, Peter Sandøe

**Affiliations:** 1 Department of Animal Science, Aarhus University, 8830 Tjele, Denmark; 2 Department of Food and Resource Economics, University of Copenhagen, 1958 Frederiksberg, Denmark; pes@sund.ku.dk (P.S.); 3 Department of Veterinary and Animal Sciences, University of Copenhagen, 1870 Frederiksberg, Denmark

**Keywords:** agricultural colleges, farm workers, animal welfare, Danish, education, perceptions

## Abstract

**Simple Summary:**

Since farmers’ perceptions of animal welfare affect the way they treat their animals, it is important to study the formation of the attitudes of future farmers and farm workers. Using individual and focus group interviews, we studied how education affects Danish agricultural students’ view of animal welfare. We also interviewed teachers at the agricultural colleges to see how they perceive the importance of animal welfare. Our results show that students, both older and younger, often see animal welfare as valuable if it can be combined with productivity. To an extent, the students also focused on the importance of having a personal, positive relationship with the animals. Older teachers indicated that animal welfare has a much bigger impact today than it did 20 years ago, and all the teachers felt it should be included across all relevant subjects. However, animal welfare is not defined in any particular way in the relevant curricula. Agricultural education ought to have an important role in teaching about animal welfare in a society where farmers are increasingly required to engage in market-driven improvements in animal welfare.

**Abstract:**

Farmers’ perceptions of animal welfare have been found to affect the way they treat their animals, and there is growing awareness of the range of ethical views today’s farmers take on animal welfare. The purpose of this study was to examine how perceptions of animal welfare develop among prospective farmers and farm workers in Denmark during their studies at agricultural colleges. We also examined the experiences of college teachers and their views on the inclusion of animal welfare in livestock courses. Drawing on individual interviews and focus group interviews at four Danish agricultural colleges, we used systematic text condensation to identify three major themes among the students: 1. The importance of balancing welfare and productivity, 2. Concerns about the animal itself, 3. Concerns relating to the students themselves. Our interviews with teachers revealed a growing awareness of the inclusion of animal welfare in Danish agricultural colleges, but also disagreements over the way animal welfare should be understood. We conclude that the education of future farmers in Danish agricultural colleges today embraces animal welfare but should leave more room in the future to introduce students to the issue of market-driven welfare and consumer interest in animal-friendly production.

## 1. Introduction

Farmers’ perceptions of animal welfare have been shown to affect the way they treat their animals [1,2,3]. It has also been demonstrated that farm workers’ attitudes to animal welfare can be influenced by the working environment on the farm [4], and there is a growing awareness of the range of ethical views taken by today’s farmers on animal welfare [5].

In their daily job, farmers and farm workers must navigate between different domains—e.g., the domain of animal welfare legislation, on the one hand, and on-farm practice, on the other—where the more individual values of farmers and farm workers are central [6]. Besides this, farmers today are expected to engage in opportunities to boost market-driven animal welfare and to be innovative in creating a more animal-friendly production. With consumers asking for specific improvements to animal welfare, the demands from society can seem to generate a number of very important but risky choices for the farmer [7].

Peer-to-peer knowledge and tradition are known to have played an important role in how farming culture is passed on [8], but another important set of institutions for its transmission are the vocational schools for farmers-to-be. While research into animal welfare to date has focused on attitudes and perceptions displayed in the course of daily work on farms, it has told us little about agricultural education and its impact on the way future farmers engage with animal welfare. Agricultural education has been an essential ingredient in the success of agricultural developments in a number of European countries [9], and with the growing demands from society for farmers to engage positively with animal welfare that education is set to be more important than ever.

In search of what she calls ‘the Urban Cowboy’, Daigle [10] argues that the increasingly urban United States population, combined with an aging population of agriculturalists, creates a need to recruit, retain and educate younger generations about animal handling and husbandry in farm animal production [10]. She also argues that much of the animal husbandry information historically absorbed through experience on the family farm must now be delivered in the classroom, and she finds that there is a need to somehow disseminate generations-old knowledge about how to care for agricultural animals. Mench [11] emphasizes that there has been a blossoming of undergraduate courses addressing animal welfare in the U.S., and that some of these courses also focus on animal ethics.

In this paper, our focus will be on the way education shapes perceptions of animal welfare among agricultural students in Denmark, but before we turn to that subject, we will briefly examine the related field of animal welfare legislation and inspection, and the farmers’ ability to comply with the goals of the legislation.

### 1.1. Animal Welfare Legislation and Farmers Ability to Handle It

In the European context, animal welfare legislation will be an important element of future farmers’ education. The use of animal welfare legislation to protect farm animals and bring improved animal welfare into husbandry practices has been central in Europe since the 1960s, both at a national level and within the Council of Europe and EU [12]. The ban on isolating calves after eight weeks, and the outlawing of conventional cages for laying hens and of confinement of pregnant sows, are examples of EU initiatives which, when translated into national legislation, have bound farmers [13,14,15]. These legislative initiatives were all initially met by protest from farmers’ organizations, but today they are largely accepted as framework conditions. However, other EU initiatives, such as the EU ban on tail docking of pigs from 2003 [16], have had lower uptake, and have encountered various barriers that farmers and other stakeholders have not been able to overcome.

Research demonstrates that it can be difficult to motivate people to change by introducing legislation and similar initiatives that involve bureaucracy, inspection and punishment. As shown by Escobar and Dermeritt [17], farmers can see the paperwork that often accompanies legislative demands as something that they are obliged to carry out for the sake of the authorities and not a task they associate with care of their animals. Therefore, farmers can see documentation requirements as a burden that they are motivated to work against [17]. In addition, studies have shown that resistance to legislation has to do with uncertainty and working with unknown systems, and that new ways of working need to be facilitated with the communication of relevant knowledge. Lauwere et al. [15] saw this when they studied farmers’ unenthusiastic adoption of the EU demand to group-house pregnant sows.

Over the past two decades there has been a growing focus on so-called “market-driven” animal welfare, where animal products with higher animal welfare provenance than the standard products are sold with special labels [18]. These products typically come at a higher price, part of which is used to pay the farmer a premium [18]. In this way, such initiatives do two things: they serve the consumer who wants products produced to stricter welfare requirements than those delivered by existing legislation and, through the higher returns, they enable farmers to deliver such products. However, their uptake depends to a large degree on positive interest, and engagement, from the side of the farmers [18].

Animal welfare legislation, and the various animal welfare labels, standards and certifications that have been introduced, are typically followed by inspection, which can be seen as a more or less permanent feature of the farmer’s life today. A Danish study [19] has shown that although farmers felt that inspections informed by animal welfare legislation were needed because some farmers otherwise would cheat, they did not necessarily think of legislation and inspection as ways of changing their own attitudes to the improvement of animal welfare. As they are introduced by outside authorities, legislation and inspection are often regarded by farmers as something foreign and hostile, with limited relevance to the way their animals are best looked after [19].

At present, there are 17 colleges in Denmark offering different levels of education to those aiming to become a farmer or farm worker. Completion of the courses for farmers or farm workers at the colleges takes between 18 months and 4 years. Half of each course is spent working on farms completing vocational training (often for several months at a time). After four years, farmers who wish to take up further education can build another year on to the basic education—e.g., including economics and/or management.

Most Danish agricultural schools used to have their own stables and sheds with pigs or dairy cattle. However, only two schools still had these in 2015 when this study was carried out. Students are required instead to complete exercises on neighboring farms during term-time. Today there are no courses specifically on animal welfare at Danish agricultural colleges, although the subject must be included in the curriculum when relevant. A textbook on the issues, first published in 1993, came out in a completely new version in 2019 [20]. This book can be used as a basis for discussions of animal welfare in the classes if teachers wish to do this.

The first time the notion of animal welfare was included as a required element of the teaching at Danish agricultural colleges was 1997. At that time, the agriculture schools had evolved from being independent, privately run institutions to gradually being enrolled, as a part of a government drive, as technical colleges. As a result of educational reforms introduced in 2008 it was decided that animal welfare should not be taught as a separate subject anymore but would be included in all animal-related subjects wherever it was found to be relevant. It now appears in various course curricula, but the way it is taught depends on the way individual teachers plan their subjects and will differ between schools and teachers.

### 1.2. Aim and Research Questions

It is reasonable to assume that the years they spend at college have an influence on students’ perceptions of animal welfare. Despite this, no studies seem to have examined the role agricultural colleges play in forming students’ views on animal welfare. Therefore, the overall aim of this paper is to contribute to a better understanding of the way vocational training in agriculture influences students’ perceptions and thereby future farmers’ and farm workers’ likelihood of taking action in relation to farm animal welfare.

The following research questions were formulated to guide the research process:

1. How do early and later career students perceive animal welfare during their studies at agricultural colleges?
2. How do teachers at agricultural colleges perceive animal welfare and the legislation connected to this area as a part of their teaching?

In our study, we conclude that the education of farmers today embraces animal welfare. In the interviews with the students, we found that they looked at the importance of animal welfare in three broad ways: (1) Animal welfare as a matter of balancing welfare and productivity; (2) animal welfare for the animal itself; and (3) animal welfare for my own sake. Our interviews with teachers confirmed a growing awareness of the need to include animal welfare in students’ education but also disagreements over the most suitable way to understand animal welfare. We conclude that agricultural education must make more room in future for course components introducing students to the issues raised by market-driven welfare and consumer interest in animal-friendly production.

## 2. Materials and Methods

The paper draws on a qualitative study conducted in 2015 involving students and teachers at four of the 17 agricultural colleges operating in Denmark. The colleges were chosen to ensure diversity of size and uptake of students from rural as well as urban areas. Two were located near urban areas. One had its own farm. Observations were made and interviews were held at each of the four colleges.

Observations were carried out in spring-summer 2015 during visits to the four colleges, with two to seven days being spent in each college. The aim of the visits was to gain a contextual understanding of students’ perceptions of animal welfare and to prepare the question guides for the interviews. During the visits the researcher stayed at the college or nearby and took part in, for instance, meals with the students, sessions in the classrooms and the stables, excursions to farms, and other activities in the students’ daily college life. Notes were taken during all observations. For example, it was observed in the classroom how students were taught pain assessment in dairy cattle, or how they were instructed to carry out behavioral assessment of the animals (dairy cows and sows) on excursions on farms, and how these assessments were discussed in the classroom afterwards.

Since the aim was to capture differences in the early and later career students’ perceptions of animal welfare, two sets of interviews with the students were carried out. First, 11 individual face-to-face interviews with first-year students were conducted. For practical reasons, these students were interviewed two to three months after starting college. Members of this group were all enrolled on basic training and had not yet chosen subjects in which to specialize. The level of practical experience with farms and animals varied among the 11 students: some had grown up on farms and/or had work experience on them, while others were from urban areas and had little or no experience with livestock. Second, three focus group interviews (six to ten students per group) with older students were carried out. The participants had all been studying for three and a half years and were in the final phase of their studies. They all specialized in either pig or cattle production. All focus groups included male and female students and students with rural and urban backgrounds. Unlike some of the first-year students, these students had extensive work experience from their vocational training on farms.

The 11 individual interviews were conducted in the colleges and involved all four colleges. The plan was to hold four interviews in each college, but in one case only three were carried out. The nine teachers also span all four colleges, with two in each of three colleges and three in one. The focus groups with older students were held in three out of the four agricultural colleges. The group interviews were conducted in classrooms at the schools to make it easier for students to participate.

All of the students were recruited by snowballing and were identified and approached by teachers at the colleges, who were asked to apply detailed recruitment criteria—e.g., variation in gender, age, rural/urban background—and also asked to ensure that the students had different experiences in working with farm animals (pigs or dairy cows) [21]. The individual interviews included sections on the students’ relations with animals and their reasons for choosing to study at an agricultural college. These were followed by sections on the students’ relation to agriculture and their perceptions of animal production, with a particular focus on the welfare of pigs and cattle. Finally, the guide included a ranking exercise concerning the so-called “five freedoms” [22].

The focus groups included sections inviting the students to rank animals and farm animals and discuss issues in animal welfare in relation to the ranking. These were followed by a section during which interviewees were asked to identify and discuss things about pig and cattle production they found problematic in terms of animal welfare. After that they were encouraged to discuss the “five freedoms”. They were then asked to recall good and bad experiences with animals from their vocational training. The interview concluded with a discussion of whether and how there are differences between early and later career students in their view of animal welfare [23].

The face-to-face interviews with teachers were conducted with nine members of staff at the four colleges (seven female, two male). The teachers were recruited from the same colleges as the students in the investigation and were selected by the first author after she had made observations at the four colleges. The criteria applied in the selection ensured that the teachers were teaching in subjects related to farm animals, had various lengths of work experience and differed in age and gender. The interviews included sections on the experience of the teachers in bringing animal welfare into their teaching, the materials they used, the challenges they saw in teaching animal welfare and how they ensured that their knowledge in the area was updated and current.

All interviews followed interview guides [21]. The three guides used are presented in Supplementary Materials. Questions were constructed by the first author together with the second author. They were based on knowledge obtained in the observations and the authors’ own knowledge of animal welfare themes. The questions were piloted by the first author, who talked to both students and teachers in one of the colleges.

In preparing the interview guides, the aim was to make interviewees comfortable during the session with, for instance, talking about their own animals and family first. The guides were also written in a way using the term “animal welfare” as little as possible at the beginning to avoid reactions to preconceived ideas associated with the term “animal welfare” and instead to bring forward the experiences of the students with animals.

It was anticipated that individual interviews would be more rewarding with the younger students as they were new to the college and unused to each other and to discussions. The older students had previously tried to discuss farm animal welfare in the classroom and we estimated that some energy and surprises would be generated by letting them meet each other in this subject. This choice was also based on discussions with the teachers.

The individual interviews lasted about one hour each. The focus group interviews lasted between one and two hours with a break halfway. The first author carried out all of the individual interviews. The focus groups were run by the second author at two schools and the first author at one school.

The subsequent analysis of the interviews follows the stepwise procedure suggested by Malterud for what is called systematic text condensation [24]. Systematic text condensation is a descriptive and explorative method for the thematic cross-case analysis of different types of qualitative data. It consists of the following steps: (1) total impression—from chaos to themes; (2) identifying and sorting meaning units—from themes to codes; (3) condensation—from code to meaning. (4) At the conclusion of this stepwise procedure, patterns in the condensations were identified and described [24].

No ethical approval was required for the study, and at the time, no Institutional Review Boards existed to which we could apply for review. Informed consent (where the aim of the study was explained) was obtained from the teachers and students, both orally and in a written text, before the interviews began. All of the interviews were recorded and then transcribed verbatim, and great care was taken to remove any information that could enable identification of individual interviewees. All names used in his paper are invented.

## 3. Results: Students

The individual and focus groups interviews showed that animal welfare plays an important role on the college courses. Thus, the welfare of livestock animals was addressed by both the teachers and the students on the different courses and was often the subject of heated debate. In trying to define animal welfare, the students exhibited two dominant points of view: some saw animal welfare as the absence of maltreatment and cruelty; others interpreted it more broadly to include needs going beyond the basic requirements of feed, water, and shelter, including animal care, the environment, and space.

Although there were some differences between the younger and older students, the most striking result was that both groups tended to justify concern about animal welfare in a similar way. When elaborating on their more general points of view and arguing for the importance of animal welfare, both younger and older students referred to three distinct kinds of justification: they supported their views either by saying that a balance needed to be struck between welfare and productivity, or with reference to the interests of the animal itself, or with reference to their own interests.

### 3.1. A Matter of Balancing Welfare and Productivity

By far the most widely shared point of view about animal welfare in both groups of students was that it was important for economic reasons since it generated improved productivity. This view encompassed arguments for animal welfare in terms of providing sufficient feed, water, space and rest, as well as ensuring minimal stress and the maintenance of good health. Paul, a younger student, argued like this:

*They do need fodder, rich in nutrition, so they do not get skinny and the farmer at the end has to put an animal down. It is expensive for the farmer at the end, so the best fodder and no mistreatment.*

Another first year student, Rie, agreed:

*It’s important with fodder and water [to give them] as much as they like. I think the pigs should always have access to that where they are placed, while the dairy cows should be able to get up and move to get access to it. It is important because without it they cannot produce.*

The typical reasoning offered here was that there is a close relationship between welfare and productivity. Thus, lower welfare standards were believed to reduce yields, while higher standards increased them. What, exactly, was a sufficient level of welfare was determined by balancing the farm’s productivity, as argued by these final year students:

*Alfred: Imagine a production system [where the dairy cows are let out]. Watch the animals and you will see that they drop when they are let out to graze. They drop in yield. And if it’s raining, they will stay inside the stables anyway. And if it’s too warm, they don’t like lying in the open field either.*

*Andy: Right, it has to be profitable, it has to be.*

Notice that the students here are searching for a balance between animal welfare and productivity, which some students—in particular, the older ones with experience from vocational training—described using real life situations. In the following remarks, Alfred explains why some of the animals at the farm where he was trained should have been put down: *[The boss says] “Let us try to leave it for the time being. Let us look at it tomorrow.” Then all of a sudden, a month has passed and she [the cow] has been walking around with a bad leg. And the farmer, he knows that if there is an inspection, well, then they will give a huge fine, and he will hardly be able to pay it.*

In this perspective, the animal is viewed as an *animal at work*, rather like other means of production. Not all animals were viewed in this way. A sharp distinction was sometimes made between pets and production animals, as noted by this older student, who had been working with mink (which until 2020 were common production animals in Denmark): *I am just looking after the animals. It is not that I’m someone who says “cute, cute, cute” … they are production animals that must work. This is a business, not a hobby. We are not meant to run around and pet them…*

### 3.2. A Dilemma in Balancing Welfare and Productivity

Among the younger students a viewpoint also emerged relating to a downside of the focus on productivity. Some students described their own internal conflicts between seeing the animal as a means of production or seeing it as “itself”. This could be seen in the remarks of the younger first-year student, Lone, when she talked about separating the calf from the cow. In one way she felt the practice was needed to obtain a maximum yield from the cow, but she also felt that the calf, with the cow, was cozy, and that this was valuable—that the calf actually has a mother.

*You do of course take the calf from the cow to get the milk from the cow (…). But I do also feel that you could do something to let the calf have extra time with the cow (…) so that they get time for bonding (…) but I also see the point in delivering milk to as many people as possible—and then we have to take the calf.*

### 3.3. The Animal Itself

Although the belief that animal welfare needs to be seen in the context of balancing welfare and productivity was by far the most common perspective, students who were opposed to this logic sometimes challenged it. One argument was that animals cannot be reduced to things—they are sentient beings and therefore have rights, including the right to freedom from suffering and to be given sufficient space to display natural behavior, etc. This position lies behind the following remarks, made by a first-year student, Maria. She sees the dilemma between her views and those we considered in Section 3.1:

*I know it may be hard that it should not just be a production animal in a sense, that “now I don’t need to look after it because it’s just a thing out there and it will soon turn into money…”*

The most common argument for the assertion that we should care for animals because of the animal itself maintains that farm animals have the need for, and right to have, plenty of room and an absence of suffering. This argument was most frequently put by the older students, who often referred to their vocational training on farms. It can be seen as a reaction to specific past experiences where the students felt that an instrumental view of animals as mere means of production prevailed.

Others, such as the older student, Anton, expressed the argument in more general terms, justifying extended care with reference to the animals (here pigs) and not the costs:

*Really, if there are no places to hide; if there are (…) fights and scuffles and…, then they are bruised and stressed. They must have places to hide…*

A few of the students noted differences between the farms and a natural situation, where animals are free to satisfy their needs and express normal behavior. The following reasoning, presented by the first-year student, Lasse, draw a parallel between the way he, as a human reacts, and the way animals do:

*The most important thing is that they have something to do (…). You need to have something to do, otherwise you will end up doing something else, perhaps something stupid. So, they need toys—they do—and something to play with, otherwise… And then they should have the opportunity to… or freedom to express normal behavior. And there has to be enough room for them to be themselves (…) so you can see that they are happy and satisfied.*

Rie, another first-year student, who had mainly worked with dairy cows, was surprised by the lack of space she saw when visiting pens with sows. She also drew a parallel between sows’ needs and those of humans:

*Really, when it comes to space, I do find that the sows have extremely little space. As humans, we like to be able to turn around and have space when we are going to lie down … but I saw how some of the sows were just not able to move their legs, because they were squeezed in their pen. I do not think that is okay.*

### 3.4. For My Own Sake

The students adopting the third position (which was also less common than the view that productivity and welfare need to be balanced) argued for the relevance of welfare with reference to themselves. For some, caring for the animals and ensuring they have a high level of animal welfare was something that made them happy. As the first-year student, Anton, put it, there is a direct relationship between how he feels and how the animals feel: *I worked at a farm where there were many hospital pens. More than recommended. I found that very nice, that there was always space and more space than needed.*

Other students—mostly younger—stressed the importance of a personal relationship with the animals. In the following comments, Lasse explains why he prefers pigs to cattle: *It’s the contact, that you can touch them. That’s what I like. It’s not the same with cattle—the only time you touch them is when milking, and then it’s only the teats. There is no human-animal contact.*

Having a clear conscience, and being able to feel good about the way you treat the animals on the farm, was stressed by some of the students. Andy related this to being allowed to put down a sick animal before it became too late and the suffering was prolonged. Additionally, he felt that working on a farm where the farmer was willing to pay you for using a little extra time on the animals gave him a clear conscience.

*Having enough time … maybe not a lot of time, but at least being allowed by the farmer to take a little extra time to check that everything is okay among the animals—I find important. And the feeling that the farmer noticed how we acted. Not just allowing us to continue to give medicine to the same pig, where putting it down would be more appropriate.*

Among the older students, the importance of direct contact was more often based on specific experiences. They often noted that a close relationship with the animals both made them feel good and had a positive function in that it made the animals more manageable.

## 4. Results: Teachers

### 4.1. Animal Welfare as Increasingly Important in Education

No specific definition of animal welfare as a *concept* was presented during our visits to the four schools. However, in the interviews all the teachers confirmed that animal welfare was given far more attention in the courses run at the agricultural colleges at the time of the interviews than they had experienced before. Christina, a teacher who had worked at agricultural colleges for 25 years described it like this:

*Earlier animal welfare was not addressed; neither in teaching nor in relation to taking care of the animals at the schools. It was not given any consideration. This has changed a lot today, where a big part of the training and education actually includes discussing for instance: What sort of animal are we working with and how do we make this animal comfortable? In a way, that it can produce what we need? If you think back at the nineties, animal welfare just did not exist as a word.*

When the teachers explained why animal welfare had become increasingly important they pointed to the following themes: Animal welfare has grown as an area for debate in society and is constantly visible in the media. It has also become a central part of the profession. Organic farming is growing, and animal welfare legislation has become increasingly important. The teachers also said that today’s students have an expectation that animal welfare will be included in agricultural education.

### 4.2. Animal Welfare and Ethics as Dependent on the Priorities of Teachers

Some of the younger teachers had never experienced a time when animal welfare was not part of the debate or the curriculum. On the other hand, they emphasized that the priority given to it today depended on the interests and priorities of individual teachers. Thus one teacher, Anna, stated:

*Of course, we have objective goals to reach in the [students’] education, but it is still up to the individual teacher how you prioritize the time. I can… choose to use more time on breeding if this is my special interest and less time on fodder, for instance. It is not described in the course plan exactly how many hours “animal welfare” should last.*

In addition, disagreement among the teachers over how animal welfare is best understood and defined was expressed in the interviews. Gry, a younger teacher, said that she herself would never be able to work on a conventional pig farm. She was uncomfortable with euthanizing pigs, which you have to be able to do in pig production. She also disliked castrating male piglets:

*Obviously, this resistance of mine does affect my education of the students. However, most of the way I teach is done in relation to traditional production. Still, I cannot help showing them loose-housed sows, for example, in films from that sort of production. I do think this will be the future.*

The teachers at the four Danish agricultural colleges interviewed for this study had diverse educational backgrounds. Some had graduated from university; others had a background as farmers. This was reflected in some discussions of animal welfare among colleagues.

Gry described a conversation she had had with a colleague in which they discussed a photo of a sow with rectal prolapse being shown during class.

*I said aloud that this (the prolapse) really looked so terrible and disgusting. Then my colleague said: “Well, that is the way it is”, and “it does not suffer”, things like that. I argued that this does not change the fact that any consumer who saw this would think this cannot be true! Some suffering we should not get accustomed to look at. However, you do. However, perhaps because I, myself, have not been in pig pens a lot, I have not become blind in the way that I see some people become.*

### 4.3. Teachers Balancing Students’ Experiences with Vocational Training

Some teachers also said that they needed to challenge some of the views the students brought back from their vocational training on farms. Sometimes the students would refer to “what used to be done” on “my farm” or parroted the voice of the farmer, or “my boss”. They had acquired beliefs that they took to be knowledge, and the teachers sometimes saw that the beliefs were not in accord with, for instance, the newer and original knowledge coming out of research. Berit, a teacher, said that when students came back to the school after vocational training, she had to remind them that animals are sentient beings:

*The students are extremely loyal to the farmers when they have been out working at a farm. They often come back to the classroom with a very black and white understanding of production. However, animals are living creatures, so you have to make them understand that the reality is something in-between. I do remember when I, myself, did vocational training, and I worked on a farm where the farmer had room for 80 dairy cows. However, he had 130 in that stable. Moreover, he could not possibly see that this was a problem. When the cows needed to lie down they would just push other cows away. We have to get the students away from this way of thinking, and this is why we, as teachers, have to influence the students from their first day at the school.*

However, another teacher also pointed out that, to some students, animal welfare is an obvious part of their education today. Thus, the students had never experienced a time where gestating sows were tied up individually. For them, sows had always been kept in group housing in accordance with the legislation. This experience had to be reflected in the courses, and subjects that could not have been discussed ten years previously were much easier to address at the time of the interview. Thus Christina, an experienced teacher, said:

*Discussing welfare and behavior is much easier today than [it was] just ten years ago. Ten years ago, it was a fight. The students are much more responsive now. In fact, some of them have very different goals in relation to welfare than the farmers they work for, and they risk feeling frustrated when working on the farms because they cannot always work the way they prefer.*

### 4.4. A Focus on Observing Animals

Some of the teachers underlined a new development that had occurred during their period of employment at the agricultural colleges: personal observation of the animals. This had once been left to the farmers, who were expected to emphasize it while the students were doing their vocational training. However, both farmers and students had increasingly pressed for the theoretical teaching to be combined with personal observation as part of the courses run in the schools. As one of the teachers, Mogens expressed it:

*This combination of theory and observation sometimes really leads to surprises among the students. We used to be more focused on, for instance, doing math exercises on the correct combination of the content of fodder, and we tended not to include looking at the animals.*

Dorte, another teacher stressed that the students needed to learn to look at the animals in a more systematic way as a part of their education in animal welfare because they had to be able to recognize abnormal behavior, and it was not enough to read about it:

*I always fear that they end up thinking of abnormal behavior as something that is normal. Because if they have worked for a long time on farms (…) and seen too much (…)—for instance, how cows lie down and how they rise—they might end up thinking: “Oh yes, this is just how a cow does this.” For instance, if it likes to stand with its hind legs outside the lying area for a long time they think, it is a nice way for a cow to stand. Then I have to tell them that this is NOT normal for a cow and (…) there are so many things that they might think are “normal” because they have seen them as ordinary in the stables they have worked in.*

## 5. Discussion

Our study set out to explore differences in the ways early and later career students perceive animal welfare during their studies at Danish agricultural colleges. We also wished to examine ways in which teachers at agricultural colleges regard the inclusion of animal welfare themes and modules in the education of future farmers and farm workers. Our results showed that both teachers and students are engaged, and familiar, with the term “animal welfare”. We also saw that although there were some differences between the younger and older students, all students tended to justify animal welfare in a similar way. The most dominant reason for maintaining good animal welfare to emerge was the argument that this goal is part of a balance between welfare and productivity where animal welfare is seen as a means of running a profitable farm.

The older teachers indicated that animal welfare is much easier to include in their teaching today than it was when they first came into teaching. In addition, students expect it to be part of the curriculum. Teachers also pointed to the necessity for agricultural schools to balance the knowledge that students bring back from farms with a more up-to-date science-based knowledge in the classroom. Still, whether animal welfare is discussed and integrated into the college courses depends on the interests and priorities of individual teachers, and we found that the teachers do not always agree on how welfare is best understood. The students’ belief that animal welfare must be secured in a balance between welfare and productivity—but also to a certain extent include the animal’s need to exercise its normal behavior—parallels what has been found in research into European farmers’ perceptions of animal welfare [25]. In an investigation of the attitudes of Dutch pig farmers to animal welfare Van Huik and Bock [1] found that farmers operating in markets that focus on price and production efficiency tend to define animal welfare in terms of health and optimal performance. However, they also found that farmers operating in markets with a broader sense of quality, which incorporate values such as naturalness, animal welfare and care for the environment define animal welfare in terms of the room the animals have to express natural behavior [1]. It is interesting that farmers-to-be share an approach to animal welfare that contrasts starkly with the views of members of the public, who typically justify animal welfare with reference to the animal itself and emphasize freedom, space and naturalness [26]. Moreover, it seems the students have taken over an idea about productivity as a sign of good welfare, even though production or yield today are widely perceived as poor welfare indicators. We are not suggesting that students should adopt the position of the public, but rather that a societal dialogue about the use of farm animals would benefit from future farmers being encouraged to understand the public’s stance, and that agricultural colleges have an important role to play in presenting students with alternative perspectives on animal welfare. Furthermore, it seems that a shift in perceptions may lie in store, as our results show that some of the students have a growing interest in the significance of space for the animals. Younger students pointed to how restricted sows are in the farrowing unit. They disliked what they saw. These observations from the students are important, as they may mean that in the future they will be interested in improving farm systems that give animals limited opportunities in terms of space and freedom to move around. In addition, if these systems, like the farrowing of sows in confinement, are not changed, the agricultural sector could lose some of these young, critical students simply because they find it hard to work with production systems they find problematic—for the animals, but also for themselves. How to make room for the animals as stakeholders and not only seeing them as ‘workers’ performing for humans under conditions that often does not really cater for their behavioral needs is an ongoing discussion in animal welfare theories [27] and we see it as relevant also to introduce this discussion in the agricultural educations.

Although agricultural education has been an important factor in the success of agriculture in Denmark and other European countries [9], the advantages of including in it the issues raised by animal welfare as a part of the education of future farmers has not been studied before. Studies of education in areas bordering agricultural education, such as veterinary training, have suggested that specific animal welfare courses, delivered, for example, online, can be beneficial: such courses make students more comfortable about educating themselves and they then score significantly higher in identifying welfare factors than students who did not take a course [28]. A study by Ventura et al. [29] focuses on the way the perspectives of veterinary students on animal welfare can change after lectures and farm visits, but quite how farmers, or farmers to-be, can benefit from specific learning in relation to animal welfare is poorly understood.

Franz et al. [8] have stated in their research that the way farmers learn relates mostly to first-hand experience and is motivated by the desire to save time and money. They add that farmers enjoy peer-teaching. In a Scottish study, Austin et al. [30] examined the attitudes of both farmers and agricultural students to animal welfare and found, for example, that students who are knowledgeable about animals tend to like animals, regard them as individuals, and will be less theoretical in their welfare attitudes.

This echoes our results, in particular the fact that some of the teachers at Danish agricultural colleges reported that the students found peer-training, and the knowledge they acquired from bosses on the farms where they had worked, very trustworthy. However, blind trust in the peer-to peer information gathered during vocational training was in our results described as a challenge by some of the teachers, who felt they had an important role in supplying the famers-to-be with knowledge based on the newest research. Daigle [10] observes that much of the animal husbandry information historically learned through experience on a family-run farm must now be delivered in the classroom. She says there is a need to disseminate generations-old knowledge about how to care for agricultural animals. We see it as a challenge for the agricultural sector that information provided by the colleges may in future be seen as less trustworthy than the understanding students pick up working on farms. We also see a challenge for the teachers at agricultural colleges to secure a proper standard for education in animal welfare, as the colleges do not have clear minimum requirement and teaching in relation to animal welfare still depends heavily on the interests of individual teachers, as shown in our results.

It will also be problematic if future farmers mainly copy older farmers’ understanding of animal welfare, on which the welfare has to connect in some way with productivity especially, since this is far from the understanding that consumers have. Today it seems that animal welfare is becoming more and more firmly linked to market-driven possibilities and less to legislation [16]. This means that future farmers must be introduced to, and become familiar with, the animal welfare views that are operative in the market and not just copy each other. Our study indicates an educational gap, as neither the teachers nor the students actively explained the significance of the market in the development of improved animal welfare.

Farmers’ willingness to change, for instance, to more animal-friendly systems could be essential to their survival in the agricultural business in the future. This means the agricultural colleges have an important role to play in presenting students with alternative viewpoints on animal welfare and preparing students to take part in a societal dialogue about the use of farm animals. We hope more studies will be conducted specifically on the role of agricultural education in different countries, in connection not only with animal welfare, but also with environmental issues and climate challenges.

This study, using a qualitative methodology, does not tell us how representative the views presented here are. It could therefore be advantageously supplemented with further survey-based studies. Nor can we generalize to conclusions about the way agricultural studies affect students in other countries. However, it seems unlikely that the general tendency we have observed, where students are inclined to follow in the footsteps of the older farmers when it comes to animal welfare, is a purely Danish phenomenon. Studies by Lave and Wenger [31] on situated learning stress that learning is not just about the teacher relationship but also happens in social relationships. In our study, we saw important conflicts between situated learning in a social practice on farms—and a rising demand for new knowledge—for instance, in relation to animal welfare learned at college in the classroom. We think the different ways of learning should be an issue of far more interest to agriculture and its educationalists than appears to be the case at present. We also think our findings can be a useful first step to further investigations in a field that seems understudied, and perhaps this will lead to more detailed studies of the ways in which farmers learn.

There are some limitations of the current study. For example, we tried to compare differences in perceptions of animal welfare between early and later career students. Ideally, we could have followed the same groups of students for 3–5 years. However, this was not possible within the framework of this study, financially and timewise.

## 6. Conclusions

Both the students and their teachers in the present study were engaged, and familiar, with the term “animal welfare”, and although there were some differences between the younger and older students, all students tended to justify animal welfare in a similar way. The dominant argument for maintaining good animal welfare was that this goal is part of a balance between welfare and productivity. Other arguments called upon in support of welfare-sensitive care for livestock referred to the animals’ needs or the students’ own interests, but these arguments were less commonly presented. It emerged that teachers play an important role in balancing what students learn in their peer-to-peer training on farms and what they learn at college. We argue that college education delivered in the classroom will play an important role in the future of agricultural businesses. We note that it will be problematic if future farmers simply copy the understanding of animal welfare that they have seen among colleagues on farms, since this is very far from the way in which consumers today understand animal welfare. Furthermore, we find that agricultural educations should be open to new challenges regarding animal welfare and invite both teachers and students to engage in discussion on how animals can be seen as not only workers for humans but also as stakeholders that must be considered in their own right.

## Data Availability

The data presented in this study are available on request from the corresponding author. The data are not publicly available due to fear of stigmatizations of interviewed students.

## Supplementary Materials

The following are available online at https://www.mdpi.com/2076-2615/11/3/696/s1, 1. Interview guide for individual students. 2. Interview guide for focus groups with older students. 3. Interview-guide for teachers.
