# Supplementary material for: For the Sake of Production—And the Animal, and Me. How Students at Danish Agricultural Colleges Perceive Animal Welfare
Source: Animals (Basel). 2021 Mar 5;11(3):696. doi: 10.3390/ani11030696 (PMC8001740; doi:10.3390/ani11030696)
Supplement: Supplementary file 1 [file animals-11-00696-s001.zip › supplementary/1. Translated Guide individual interviews, younger agricultural students and animal welfare.docx]

| **Purpose: Individual interviews with students** | **Method and comments/facilitation** | **Questions/key words** |
| --- | --- | --- |
| 1. **Introduction (5 minutes)** |  |  |
| Introducing myself, the topic, the interview form and giving other practical information | Our research is concerned with the way agricultural colleges teach when it comes to farm animals.We think the colleges are important in the education of future farmers and farm workers – and because there is a lot of discussion about farm animals we would like to learn more about the way the colleges teach by talking to you, the students. | I am from Aarhus University where I am working on research in future farmers and their relation to their animals.  My name is Inger and I will be doing the interview, which will last about an hour and a half.  It is important to bear in mind that there are no wrong answers: I am here to listen to your thoughts, opinions and attitudes, not to check up on factual knowledge.  *.* |

| 1. **Background (5 minutes)** |  |  |
| --- | --- | --- |
| The participants introduce themselves.  Purpose: to create a pleasant and informal atmosphere. To obtain background information about the student, without mentioning animal welfare! |  | I would like to tell me where you come from, what your interests are and why you chose to go to an agricultural college.  Age   - Your background in farming – your interest in farming – the environment you come from - How have you experienced the change from living at home to staying at the college - Social life at college - hobbies |
| The purpose is to examine the role animals have had in the student’s life up until this point, to what extent they have been/are concerned with animals as pets or production animals and what values they think are important in connection with animals.  The aim is also to see if the students put forward the concept of animal welfare of their own accord in a general discussion about animals.  The interviewer must not introduce the concept of animal welfare. | The animals in their lives – individual round.  **Facilitation:** Questions relating to the actual relationship between student and animals. | - Can you say something about how much you have had to do with animals before you started at agricultural college? - How did your interest start? - How much time did it take up up in your daily life? What was your relation to the animal? - Is there a difference between this animal/these animals and farm animals? - If the relation in childhood was to farm animals, were there also pets in the student’s life – was there a difference in the relation? - Are there animals you do not like? – give examples. |
| Animals at the college and in farming (5-10 minutes) The purpose is to examine the attitude to animals in the context of the course of their studies and the agricultural business. Thematically we want to start with the course (the student’s personal experience) and then go on to the use of animals in farming (depending on the student’s background/experience they might have actual experience or ideas about farming.)  Again, the interviewer must not use the concept of animal welfare, as we want to see if the students themselves use the term in the discussion of animals in these two contexts. | **Facilitation:**  Questions relating to the student’s motivation for choosing to work with animals at college.  Questions relating to what they expect to learn about animals during their course.  Introduce the upcoming work experience to guide the dialogue on to actual farm animals or draw on earlier work experience for example with a neighbour.   - Start explanatively by asking why they chose to study at an agricultural college to start with, if it has not already been answered in the introduction. You may repeat the question: you chose the agricultural college because you father is a farmer…/because you love horses... | - Say something about why you chose the agricultural college - Are you going to stop after the first year course so you become an assistant – or are you going to carry on and become a trained farmer? Why? - Expectations to what animals you will be working with after the first year course ( where they choose a specific farm animal to work with for the first time) - Where are you going to do your work experience (and if relevant: why this type of farm animal? - What do you think about animals in farming? Present experience/problems relating to looking after them. - Have you had surprises during first year course in relation to your work with animals? (For example when visiting farms) Have you seen something unexpected? - Are there any areas where you think farming has particular problems when it comes to farm animals? - What will be important for you as a future farmer when it comes to animals? |

| 4. Animal welfare and farm animals (10-20 minutes) The aim is a focused discussion of the students’ perception of the welfare of production animals.  We both want to examine the importance of animal welfare when it is put forward as a central topic and what aspects (as for instance pain, natural habitat and behavior, access to food and water etc.) they think are contained in the concept of animal welfare, and finally what values the students associate with the various aspects. | Facilitation: The topic starts in a relatively explorative mode where the student is asked to do a free association exercise where he/she is asked to associate on the term animal welfare within the context of animal production. | **What is important for animals to have a good life?**  The student is given a couple of minutes to collect his/her thoughts before they explain – they are prompted to ensure clarification of the key words (write them down on paper, ask the student to elaborate).  This is followed by an open question as to which animal welfare aspects are particularly important when it comes to farm animal production (you could refer to the animal the student prefers – possibly by letting the student prioritize what he/she wrote above)   - What do you think is particularly important when it comes to the welfare of a pig/a cow/a mink? Give examples. - Elaborate you statement/ say a bit more about ….. - If a particular aspect is important, how does that fit in with the daily production? |
| --- | --- | --- |
| The next part of this topic is even more focused. The students are interviewed about their understanding of specific aspects of animal welfare with the point of departure in The Five Freedoms.  We want to examine if there is a connection between the perceptions of animal welfare that we have introduced to, based on experience or purely attitudinal, and the view represented by The Five Freedoms. | - Cards with The Five Freedoms, one on each card. | The interviewer reads out The Five Freedoms and asks the student to comment on them and organize them:  What do you think of the different freedoms and how would you organize them if you were to place them according to what is important for the animal? |
| 5. Animal welfare i teaching (5-10 minutes) The aim is to get an idea of how the student has seen/experienced animal welfare as part of the teaching.  We want to examine if the students experience ‘shortcomings’ in the way they are taught or whether they are content with what they learn and see everything as relevant.  Is there for instance room for a discussion about animal welfare? How does this discussion take place? |  | You have various subjects in the first year course from workplace environment, first aid, exercise driving tractor etc.; but you also learn about animals, both cattle and pigs (you are also introduced to chickens and mink, and sometimes-even sheep).  Can you give examples of any teaching of animals in the first year course it may be something that has made a special impression on you.  We have talked about animal welfare earlier in relation to The Five Freedoms (e.g natural habitat and behavior, diseases) Have you come across these aspects in the teaching in the first year course. How?  Is there anything in the teaching that has been particularly good in relation to the animal you have chosen to work with when you continue on the main program after the first year course? In what way do you think you have increased your knowledge in relation to your choice and the relevant animal?  Have you lacked anything in the first year course when it comes to knowledge about farmed animals? |
|  |  |  |

| **6. Concluding** |  |  |
| --- | --- | --- |
|  | Debriefing. Clarification of what the student has taken part in. Thanks and giving out cinema tickets, | Debriefing (2-5 min) Thanks for your help. A couple of words about what the student has contributed to and why we are interested in animal welfare.  What was it like to take part?  Did anything surprise you in our questions?  Are we missing anything in your opinion? |
