# Supplementary material for: For the Sake of Production—And the Animal, and Me. How Students at Danish Agricultural Colleges Perceive Animal Welfare
Source: Animals (Basel). 2021 Mar 5;11(3):696. doi: 10.3390/ani11030696 (PMC8001740; doi:10.3390/ani11030696)
Supplement: Supplementary file 1 [file animals-11-00696-s001.zip › supplementary/2 Translated Guide_focus groups,older agricultural students and animal welfare.docx]

| **Purpose: Focus group interviews with older students at agricultural colleges** | **Method & Comments** | **/keywords** |
| --- | --- | --- |
| 1. **Introduction (5 minutes)** |  |  |
| To introduce ourselves, the theme, the type of interview, and to give other practical information | Our backgrounds, and the theme  Remember to give our names | *We are from Copenhagen and Aarhus universities*  *where we are doing research on future farmers, and their attitudes to farm animals*  *My name is Jesper, and I will be chairing the discussion. I will give you some examples to work on and things for you to discuss. The idea is for you to discuss with each other – so speak up if you disagree or have something to add*  *Remember – there are no wrong answers: we are here to listen to your ideas, opinions and attitudes, not to test how much you know.*  *It should take about two hours, and we will have a short break after about an hour*  *Inger, here beside me, will look after the equipment, and will not be saying much. As you can see, we will be recording the session, but you will be anonymised when we refer to you*  *It is important that you explain what you have done while working on the examples, so we will understand better what has been going on when we listen to the session* |

| 1. **Background (5 minutes)** |  |  |
| --- | --- | --- |
| Participants introduce themselves, so we all get an idea of each other. Everyone will have a chance to say something to create a sense of trust | A – individual introductions | *Let’s start by introducing ourselves. Please say where you are from, your interests, and why you have chosen to go to agricultural college. Please remember to say your names, so those writing out the transcripts know who is who.*   - name - background (town or countryside) - hobbies - Why agricultural college? |
| To get a sense of what experience participants have had with animals, and what role these have played in their lives. | 1. You and animals – individual participants | *Let us talk a little about what experience with animals you had before starting college. I mean both farm animals and pets, or hunting or fishing* |
| 1. **Animals in general** |  |  |
| Finding out what participants think about animals in general, their personal attitudes to them, and how they talk about animals  To get an idea of how the participants prioritise the animals, and what arguments they use to explain this, including how animals are given different values.  To get a sense of how farm animals get put in a different category to other animals | **Group session – Animal hierarchy**   1. highest priority   Participants are given – one at a time – cards with the names of animals in different categories which they are asked to prioritise   - pigs - cattle/dairy cows - dogs - cats - rats - snakes - apes/monkeys - horses   **NB take a picture**   1. Importance   If someone asks what ‘important’ means, they must decide that among themselves | *We are going to talk a bit about animals in general – not just farm animals. I have some cards with the names of various animals. I will give you these one at a time, and ask you to lay them in order so the animal you think should be treated best is at the top*  *Remember that you have to agree, so you need to discuss in what order the animals should be put*   - Why have you put the animals in this order? - Why is X higher than Y? - What is the difference between…?   *What if I ask instead which animal is the most important – do any need to be moved?*   - Why ought X be moved up/down? |

| 1. **Farm animals** |  |  |
| --- | --- | --- |
| To get a sense of what the students themselves think is important when ranking various farm animals | **Group session**  Exploratory exercise: Farm animals grouped according to self-chosen criteria  Animals on cards   - dairy cattle/cow - beef cattle - egg laying - broilers - sows - Finishers - mink - bull   NB: If the animals are grouped according to species, repeat the exercise allowing only two groups | *For the remainder of the time we are going to talk about farm animals, and you will get some new cards with typical Danish farm animals*  *Please look at the cards first, and then try to put them in 2-3 piles of animals* ***that you think belong together***   - *Could you make other groups?* - *What if you are only allowed to make two groups?* |
| To find out what criteria the students think are relevant for animal welfare for farm animals, and discover general attitudes to this based on the students arguments | **general rank order exercise**   1. according to consideration/respect/dignity/value | *Now we need to try to put the animals in order according to how much consideration they should be given* |
| *We’ is not defined. In the next section it will be farmers, and we can try to play with the question of is it themselves or ‘society’! | 1. **Case: pigs**   Firstly exploratory. Values associated with pigs and animal welfare. Each person writes a list of keywords to be presented to the group. Then structured, with cards (and possible extra cards) grouped jointly into three groups according to importance.  Suggest three cards, if the following are not included:   - Loose housed sows in the farrowing unit - Requirement for straw as rooting -activation material - piglets remaining with the sow longer than the minimum 21 | *It is time to talk about pigs. I want you first to think about animal welfare in relation to pigs.*  *What does it make you think of?*  *What do you think is important to ensure good animal welfare for farmed pigs in Denmark*  *I will give you a couple of minutes to write keywords on the blank cards – just a couple of words on each card*  *What have you put?*  *First clip all the cards you think mean the same thing together*  *Then sort the cards into three piles in order of importance* |
| To find out attitudes, when the discussion is framed in relation to a specific animal | 1. **Case: Dairy cows**   as for pigs  Suggest three cards, if the following are not included:   - Access to pasture - Cow and calf together for 12 hours - A ban on rutine tube feeding raw milk to calves | *? It is time to talk about cattle. I want you first to think about animal welfare in relation to cattle and milk production What do you think is particularly important to ensure good animal welfare for dairy cattle in Denmark*  *I will give you a couple of minutes to write keywords on the blank cards – just a couple of words on each card*  *What have you put?*  *First clip all the cards you think mean the same thing together*  *Then sort the cards into three piles in order of importance* |
| **Break** |  |  |

| 1. **Of animal welfare during work experience** |  |  |
| --- | --- | --- |
| The aim is to link the students’ knowledge of and attitudes about animal welfare with what they have seen during work experience placements to look more closely at what effect their studies have | The students get two minutes to write about one good, and one bad, example of animal welfare from their work experience. They write on a red and a green card, which they then present to the group | *Think back to your work experience – you can choose which one you like. Try to focus on one good and one bad animal welfare example while you were there. It could be something you thought was bad and needed changing, or something that was good to see that everyone ought to do*  *You get one red and one green card. Write keywords about the good animal welfare on the green card and the bad on the red card. You can have a couple of mimutes to notw down your keywords*  *Now we will go round the group – first the good example...*   - *How can these good examples be encouraged everywhere?* - *Who should ensure this happens?*   *Now the bad examples...*   - *Do you agree?* - *Do you recognise them?* - *What did you do when you saw these? Wat do you think you ought to do?* - *Did you get any help to deal with these bad examples? Who? the college?your parents?friends?*   **Exploratory qquestions**   - Has your work experience made you think in particular about animal welfare? - Do you talk to anyone about what you saw?   Did college prepare you to see things like this? |

| 1. The five freedoms |  |  |
| --- | --- | --- |
| The aim is to introduce the students to specific definitions of animal welfare that come from ”outside”, and see how the students react to them  We will try to see if there is a link between the attitudes to animal welfare that we have been presented – based on the students experience and own attitudes – and the picture of animal welfare represented by the five freedoms | The students are given cards with the five freedoms   1. **1. Freedom from hunger and thirst: by ready access to fresh water and a diet to maintain full health and vigor.** 2. **2. Freedom from discomfort: by providing an appropriate environment including shelter and a comfortable resting area.** 3. **3. Freedom from pain, injury or disease: by prevention through rapid diagnosis and treatment.** 4. **4. Freedom to express normal behavior: by providing sufficient space, proper facilities and company of the animal’s own kind.** 5. **5. Freedom from fear and distress: by ensuring conditions and treatment, which avoid mental suffering.**   Cards with all the freedoms are given out (WITHOUT NUMBERS) to everybody.  They are read out and questions asked to check understanding. The moderator should avoid explaining too much to the students. The remaining cards for each freedom are then rank ordered according to importance | *As we have already discussed, animal welfare can mean many things. When rules are made, these are often based on the ”five freedoms”*  *Have you heard of them? Can you name any of them?*  *We are going to look at them together, one at a time, and here are cards with them all so you can see them*  The freedoms are read aloud one at a time: the students are questioned about whether they understand them, and if they think any aspect of animal welfare is missing  *I would like you to put them in order, with the most important at the top, and we will start with ”freedom from hunger and thirst”*  *How is this important?*  *Why? Why not?*  The students then get cards one at a time which they are asked to discuss and put in rank order |
| 1. **Earlier and now, and what college has meant** |  |  |
| The aim is to have a concluding, summarizing discussion, that looks again at the link between attitudes to animal welfare and the effect of going to agricultural college  The aim is to get the students’ own evaluation of ”before and now”(before college and now?) What do they feel has changed them with regard to animal welfare – has the college contributed to any change or not? | Two minutes time out, where the students can write their thoughts about what they have got from their classes – or what they have thought was missing – and whether the college has affected their views on animal welfare and/or the importance of animal welfare.  (Use printed cards that show what they should note down)  Group discussion based on keywords and contributions from the students | *Lastly, we would like to talk a little about the agricultural college We would like to hear if you think being at the college has had an effect on you attitudes to animal welfare*  In other words – has your attitude to animal welfare changed since you started at the college?  *Can you give an example of something that has been particularly important for this – e.g. a class, a particular teacher, your classmates, or something else?*  ***Exploratory question***   - Is there anything you think has been missing concerning animal welfare – particular information or experience you think are lacking? |
| 1. **Conclusion** |  |  |
|  | Debriefing. Explanation of what the students have just taken part in. A Thank you to them, and distribution of free cinema tickets. |  |
