# Supplementary material for: For the Sake of Production—And the Animal, and Me. How Students at Danish Agricultural Colleges Perceive Animal Welfare
Source: Animals (Basel). 2021 Mar 5;11(3):696. doi: 10.3390/ani11030696 (PMC8001740; doi:10.3390/ani11030696)
Supplement: Supplementary file 1 [file animals-11-00696-s001.zip › supplementary/3. Translated - Guide_individual interviews, tearchers at agricultural colleges.docx]

| **Purpose: Interview with teachers at agricultural colleges** | **Method and comments/facilitation** | **Questions/key words** |
| --- | --- | --- |
| 1. **Introduction** |  |  |
| To introduce myself, the topic, the interview form and to give other practical information. | Our research is concerned with the way agricultural colleges teach when it comes to farm animals  We think the colleges are important in the education of future farmers and farm workers – and because there is a lot of discussion about farm animals we would like to learn more about the way the colleges teach by talking to you, the teachers at the colleges. | I am from Aarhus University where I am working on research in future farmers and their relation to their animals.  My name is Inger and I will be doing the interview, which will last about an hour and a half.  It is important to bear in mind that there are no wrong answers: I am here to listen to your thoughts, opinions and attitudes, not to check up on factual knowledge.  *.* |

| 1. **Background (5 minutes)** |  |  |
| --- | --- | --- |
| ´The participants introduce themselves.  Purpose: to create a pleasant and informal atmosphere. To obtain background information about the teacher. |  | - Age/educational background/are you a farmer yourself - Why teach at agricultural college today? (How long? Different types of colleges.) - How do you feel about working within the agricultural college system, anything in particular you find important in your job - Your subjects at the college – and the levels you teach at. |
| 1. **Teaching about animals – experience and materials.**   The aim is to get the teacher to talk about the animals he/ she teaches about and the problems in teaching about these particular animals. | Facilitation:  Questions relating to the teacher’s actual experience in teaching. | - You teach about xx (pigs/cattle), what do you think is most important to teach about when it comes to these animals? - How has the teaching about these animals developed in the time you have been teaching? - What materials are you using to teach about these particular animals? - Where do you get the materials? - What do you think is the most important element when it comes to teaching about animals? - Is there a difference in what is most important, when you think of the different levels of the course? |
| 1. **Teaching animal welfare**   The aim is to examine how animal welfare is taught – firstly the teacher’s understanding of animal welfare has to be clarified, secondly how animal welfare is integrated in the teaching. | Write down notes to each point. | **Step 1 (the teacher’s attitude to animal welfare)**  What is important to you when you teach animal welfare?  (How do you prioritize? Examples of how animal welfare is included)  What do you yourself associate with the term animal welfare?  The teacher’s associations and reasons for the points he/she makes.  **Step 2 (Methods in teaching about animal welfare)**  Can you explain how you plan the teaching of animal welfare when no specific lessons are allocated and the area, therefore, is not seen as a subject in itself?   - Is there a difference in the way you work with animal welfare at the first year course and and the twomain programmes ? - Can you think of one or more undervisningsforløb that you think have been particularly successful? Why? - Can you give an example of something that did not work? Why? - What materials do you use when you teach about animal welfare during the course? - Do you think the materials available are good enough? Why? - Is anything missing? What and why? - Rules and regulations/demands in relation to animal welfare – are they part of the teaching?In what way – and how do the students react when they are being taught in it? - Is there anything the students are particularly interested in, when you talk about animal welfare? If yes, why do you think that is? Do you find the students disagreeing on animal welfare? What do they disagree about? |

| 5. Students and animals The aim is to see what problems the teacher experiences in relation to teaching about farm animals. – based on the background of the students at the agricultural colleges. The questions seek to uncover if there is a new type of students that the teachers have to allow for when it comes to teaching farm animals. Have the teachers changed their goals and methods because, for instance, the students have become more critical, to a larger extents grew up in cities or are female students with a background in horse riding etc? |  | - What is the group of students like now in relation to earlier (more girls? More city kids? How do you know if the students have changed? Are there still a lot of fathers’ sons?) - If there is a particular change, does that give new opportunities or new problems when teaching about animal welfare in particular? - Particular problems in relation to the students now and earlier – also in relation to teaching about animal welfare? - Describe educational approaches being used when teaching about animal welfare. - What is your experience of the students’ attitude to animals – before and now? |
| --- | --- | --- |
| Animal welfare during vocational training at farms The aim is to examine the teacher’s contact with the student during work experience – specifically what sort of tasks they are given while they are doing work experience. The intention is to clarify if animal welfare is transferred from the college to the practical work. |  | - Who is responsible for the work experience period? Is it the college or is it the farm owner? - What sort of contact is there between college and student during work experience? ( Give examples, are they given tasks to perform?) - Do you prepare the students in relation to animal welfare before they start work experience – how? - How is work experience part of the teaching about animal welfare. Examples - Do you see a change in the student’s perception of animal welfare before and after the work experience? What happens when the students return from their first period of work experience? - Is student experience from home or workplace sometimes seen as wrong when it comes to animal welfare? Examples? - How do you as a teacher deal with a bad farm owner that you think has taught the student something wrong in relation to animal welfare? |
| 6. Debriefing Thanks for your help. A few words about what the teacher has contributed to and why we are interested in animal welfare.. |  | What was it like to take part? Did anything surprise you in our questions? Have we missed out on anything? |
